# Cancer mutations in RAD51 and its paralogues

**DOI:** 10.1371/journal.pone.0349105

**Published:** 2026-05-14

**Authors:** Anna L. Valentine, Isabella L. Huth, Nika M. Duff, Aisha Z. Rabbani, Kateri N. Donahue, Wesley A. Bush, Renee A. Bouley, Ruben C. Petreaca

**Affiliations:** 1 Biology Program, The Ohio State University, Marion, Ohio, United States of America; 2 Biochemistry Program, The Ohio State University, Marion, Ohio, United States of America; 3 Neuroscience Program, The Ohio State University, Marion, Ohio, United States of America; 4 Cancer Biology, The James Comprehensive Cancer Center, The Ohio State University, Columbus, Ohio, United States of America; 5 Department of Chemistry and Biochemistry, The Ohio State University, Marion, Ohio, United States of America; 6 Department of Molecular Genetics, The Ohio State University, Marion, Ohio, United States of America; University of Hawai’i at Manoa, UNITED STATES OF AMERICA

## Abstract

The RAD51 recombinase is central to repair of DNA damage arising from stalled or collapsed replication forks and DNA double strand breaks. Its essential role is revealed by the fact that this function evolved in bacteria but was retained in eukaryotes. In humans some of the RAD51 functions have been relegated to several paralogues which evolved by gene duplication. In addition to mutations, most cancers are also characterized by increased chromosomal instability manifesting as translocations, deletions, insertions, and other more complex forms of chromosomal re-arrangements. Given the central role of RAD51 in protecting against chromosomal instability it stands to reason that RAD51 mutations that alter its function should register in cancer cells. However, pan-cancer analyses of analyzed cancer genomes show a marked absence of RAD51 loss of function mutations leading to a so-called “RAD51 paradox”: increased chromosomal instability despite normal RAD51 function. One hypothesis is that mutations in the RAD51 paralogues may contribute to the genomic instability, meaning that a lack of mutations in RAD51 may be compensated by an increase of mutations in the paralogues. We queried analyzed cancer genomes from COSMIC and mapped all mutations in RAD51 and its paralogues. This revealed an increase in RAD51B, RAD51C and RAD51D paralogue mutations in human cancers. We used established algorithms to determine the probability that any mutation may affect enzyme function. Although, we did not find many “driver” mutations, numerous paralogue mutations were pathogenic or likely to destabilize enzyme function. In silico 3D structure analysis was then used to analyze the potential effect of some of these mutations on protein structure. Gene expression analysis did not reveal any changes in paralogue expression levels. Further, an evolutionary analysis did not uncover any selective pressure for mutations in RAD51 and its paralogues. A comparison of mutations reported on COSMIC with those reported on ClinVar revealed that many mutations primarily in RAD51C and RAD51D are also hereditary. Thus, it appears that an apparent low level of RAD51 mutations in cancer cells is compensated by an increase in paralogues mutations.

## Introduction

Homologous recombination (HR) evolved to facilitate replication of long genomes [1]. The replication machinery can stall or even produce chromosome breaks which increase genomic instability [2–4]. The HR machinery both rescues stalled replication forks and repairs DNA double strand breaks (DSBs). One clue to the essential function of HR in DNA replication is the fact that it evolved in bacteria and was retained in eukaryotes [1,5]. Another clue is that certain recombination and replication genes may have evolved from the same ancestor through gene duplication [6,7].

Central to recombination is RAD51 (recA in bacteria, also known as RAD51A in humans) which can nucleate single stranded DNA produced by resection of DSBs or stalled replication forks [8–11]. ATP binding induces a conformation change in the enzyme that increases DNA affinity, promotes filament formation, homologous pairing, and strand exchange [12–15]. In yeast, RAD51 is loaded unto the ssDNA by RAD52 [16] but in humans and other higher eukaryotes this function has been replaced by BRCA2 assisted by BRCA1 and PALB2 [17,18]. RAD52 also exists in humans but its function has been relegated to an accessory recombination pathway (single strand annealing) [19–21].

Eukaryotes have several RAD51 paralogues that assist with its function [22–24]. In humans these paralogues form two distinct complexes RAD51B-RAD51C-RAD51D-XRCC2 (BCDX2) and RAD51C-XRCC3 (CX3) [22,25–29]. BCDX2 functions to restrain fork progression under stress such as during low nucleotide pools while CX3 facilitates fork restart [30–32].

Mutations in the DNA damage response pathway (DDR) including components of the recombination machinery are common in cancer cells [33,34]. Remarkably, although RAD51 mutations register in cancer cells, none appear to be inactivating or promote tumor formation leading to the so-called “RAD51 paradox” [35]. A hypothesis explaining this paradox is that fast replicating cancer cells require RAD51 to deal with the ensuing replication stress [17]. A clue to this premise is that cancer cells appear to be characterized by over-expression of RAD51 [36,37] and inhibiting RAD51 is a therapeutic approach [38]. Too much genomic instability can kill even cancer cells and RAD51 function is required to “re-stabilize” tumor cells.

Inactivating RAD51 paralogue mutations increase chromosomal instability [39] and promote tumorigenesis [22,40–42]. Even certain paralogue polymorphisms appear to be associated with an increase in predisposition to some cancers, primarily breast and ovarian [43,44]. Other RAD51 paralogue mutations can also increase survival of cancer cells [45,46]. Thus, it appears that a lack of inactivating RAD51 mutations in cancer cells is compensated by mutations in its paralogues.

In this report, we used the Catalogue of Somatic Mutations in Cancers (COSMIC) database [47] to characterize all mutations appearing in RAD51 and its paralogues. The goal was to map all mutations and identify the degree to which they affect gene function. Our analysis shows that an apparent lack of inactivating mutations in RAD51 may be compensated by an increase in mutations in its paralogues, primarily RAD51C and RAD51D. Thus, in cancer cells, the HR repair machinery may be destabilized by mutating RAD51 accessory factors.

## Materials and methods

### 2.1. Data accession

Mutation data from COSMIC (https://cancer.sanger.ac.uk/cosmic/) was downloaded as.csv files and analyzed in Excel on June 9, 2023. These data are publicly available and are anonymized so that the authors do not have access to information that could identify individual participants during or after data collection. These files have information on mutation coordinates as well as the cancer type where mutations occur. Data from NCBI ClinVar were downloaded on February 16, 2026 as.csv files and analyzed in excel. Mutations between COSMIC and ClinVar files were compared to see how many somatic mutations are similar to germline.

### 2.2. High frequency, driver, and destabilizing mutations

We defined high frequency mutations as those that occur in 5 or more patients. Several publications analyzing pan-cancer mutations suggest this interpretation [48,49]. Driver mutations were analyzed using the OpenCravat pan cancer CHASMPlus algorithm [50,51] which is available as web interface here: https://www.opencravat.org/. Mutations with a probability below 0.05 were analyzed. Three other algorithms were also used: Mutation Assessor [52], SIFT [53], and VEST4 [54]. These analyses are shown in S1 Table. Mutations were considered significant for SIFT if the probability was below 0.05 and if they had a high rank score for Mutation Assessor. These mutations were graphed in S2 Fig. For VEST4, the mutations are interpreted as mildly pathogenic if the score is between 0.764–0.861, moderately pathogenic if the score is between 0.861–0.965, and strongly pathogenic if the score is above 0.965. Because VEST4 produced so many more potential pathogenic mutations, we graphed only those with a score above 0.9 in S2 Fig. Please note that for RAD51, all mutations are shifted by one coordinate (e.g., R178 on COSMIC is R177 on OpenCRAVAT output). The interpretation of the mutation is not changed, only the position. This is due to a different isoform that OpenCravat uses to output variants. For all the other genes the positions correspond with COSMIC coordinates.

### 2.3. Lollipop graphs

Lollipop graphs shown in S2 Fig were made with cBioPortal MutationMapper (https://www.cbioportal.org/visualize) [55–57].

### 2.4. Sequence alignment

For the data in Fig 2B, protein sequences were downloaded from NCBI (https://www.ncbi.nlm.nih.gov/) and aligned with ClustalOmega (https://www.ebi.ac.uk/jdispatcher/msa/clustalo).

**Fig 1:**
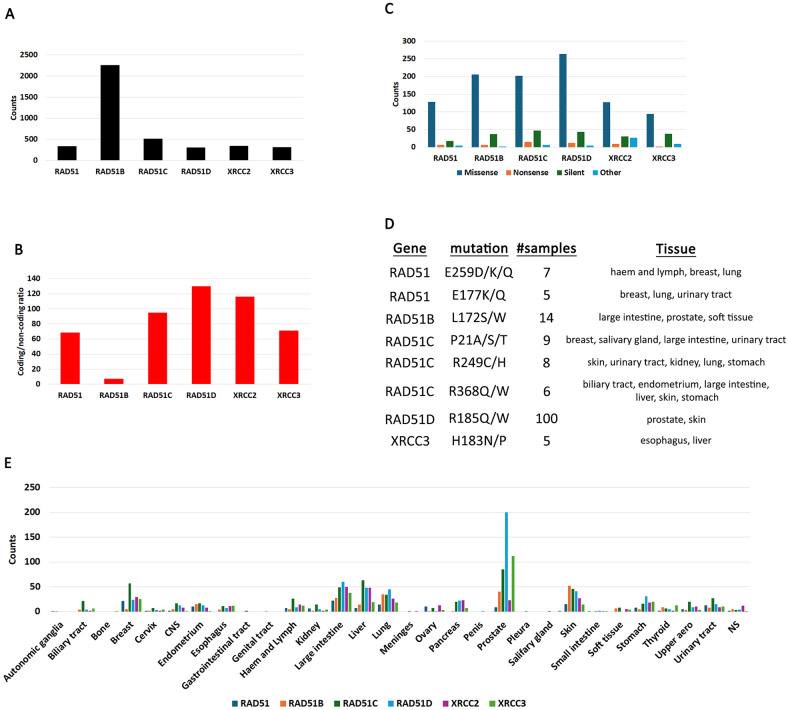
Mutation demographics in RAD51 and its paralogues. **A**. Total samples reported on COSMIC. For this graph only unique samples are reported. Multiple mutations in the same sample were counted as 1. **B**. Ratio of coding/non-coding mutations. **C**. Frequency of different types of coding mutations. Coding mutations were partitioned into missense, nonsense, silent and other (InDels, frameshift and complex). **D**. High frequency mutations appearing in RAD51 and its paralogues. These are mutations that appear in five or more samples. **E**. Cancer distribution of coding mutations in RAD51 and its paralogues.

### 2.5. Mutation co-occurrence

To identify co-occurrence of mutations between RAD51 and its paralogues, the data from COSMIC was parsed by hand. We identified those samples that had mutations in RAD51 and at least one other paralogue. The probability of mutation co-occurrence was also calculated using the cBioPortal mutual exclusivity calculator [49,55] (https://www.cbioportal.org/). This analysis was done for the pan-cancer “Curated set of non-redundant studies”.

### 2.6. Protein structure modeling of high-frequency mutations

PyMOL (version 3.1, Shrodinger) was used to generate structural models of mutations using the Mutagenesis function, identify polar interactions, make measurements, and generate figure images. The APBS software suite was used to generate electrostatic surface potentials of the wild-type and mutant structures [58]. The CUPSAT webserver was used to calculate the ΔΔG values and predict protein structure stability [59]. Experimentally determined protein structures were used for mutation modeling when possible. If experimental structures were not available than AlphaFold models were used [60]. AlphaFold models were used in all cases for CUPSAT calculations.

### 2.7. Gene expression data

Expression data for The Genome Cancer Atlas (TCGA) samples were also downloaded from COSMIC. Gene expression data are normalized to non-cancerous tissues and expressed as Z-values. These values are interpreted as normal expression (−2 > Z > 2), under-expressed (Z < −2) and over-expressed (Z > -2) [61].

### 2.8. Calculation of evolutionary selective pressure

We used a method described by Zhou et al [62]. We followed the same procedure described in our previous publication [63]. Chi-square score was calculated by hand and probability values were extracted with online calculator (http://courses.atlas.illinois.edu/spring2016/STAT/STAT200/pchisq.html) with one degree of freedom.

## Results and discussion

### 3.1. Distribution of mutations in cancer cells

When we queried COSMIC mutations in RAD51 and its paralogues, we identified that about five times more samples are reported for RAD51B than for the other genes (Fig 1A  and S1 Table). Mutations can occur in both coding regions (e.g., translated into protein) and non-coding regions (e.g., introns and 5’ and 3’ UTRs). When we queried mutations appearing in RAD51 and its paralogues we found an increase in both coding and non-coding mutations in RAD51C, RAD51D and XRCC2 compared to RAD51 (Fig 1B). RAD51B had a much higher non-coding to coding ratio while XRCC3 was similar to RAD51. Thus, most RAD51B samples do not have coding mutations (S1 Table).

**Fig 2:**
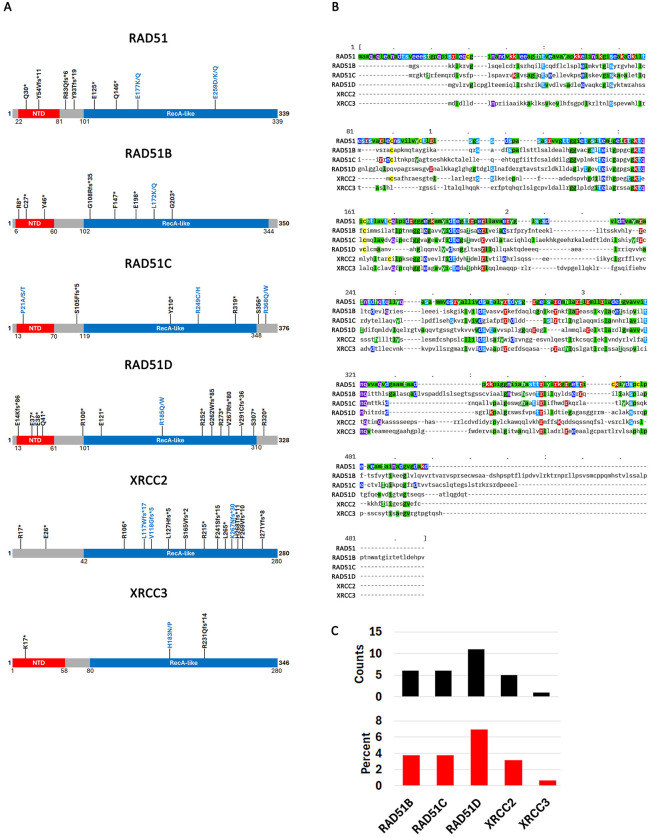
Frameshift, nonsense and high frequency point mutations RAD51 and its paralogues. **A**. Position of frameshift, non-sense (black) and high frequency (blue) mutations on cartoon diagrams of RAD51 proteins and its paralogues. Diagrams not drawn to scale. **B**. Protein sequence alignment of RAD51 and its paralogues. This alignment was performed with protein isoforms on which mutations were mapped in **A**. RAD51 (NP_001157741); RAD51B (NP_001308750); RAD51C (NP_478123.1); RAD51D (NP_001136043); XRCC2 (NP_005422.0); XRCC3 (NP_001093588.1). Please note that coordinates are given for the RAD51 gene only (top sequence).. Percent chance of a mutation in RAD51 co-occurring with at least one other of its paralogues. For example, there is a 3.79% chance that a mutation in RAD51 co-occurs with another mutation in RAD51B. Only coding mutations are considered here.

We next partitioned mutations by missense, non-sense, silent or other. Although missense mutations could destabilize the enzyme function, nonsense mutations are more severe because they all result in a truncation. The “other” category includes frameshift mutations which often also introduce a stop codon and result in a truncation. This analysis shows an increase in missense mutations in RAD51B, RAD51C and RAD51D but not XRCC2 or XRCC3 when compared to RAD51 (Fig 1C). Longer genes have a tendency to register more mutations than shorter genes and this has sometimes led to false positives: a longer gene appears to be ultra-mutated because it has more residues [64]. However, this does not appear to be the case with RAD51 and its paralogues because all genes are about the same size (Fig 2A). Thus, we interpret these data to mean that the RAD51 paralogues are more likely to be mutated. Remarkably, truncating mutations which are almost always inactivating are not increased in the paralogues. This suggests that even in the paralogues, most mutations that may affect enzyme function are point mutations.

We next looked for mutations that occur in 5 or more samples which some metagenomic studies have suggested that they should be interpreted as frequent [48,49,65]. We are cognizant that this interpretation is somewhat subjective, but we nevertheless chose this analysis hoping to uncover some hotspots. We identified several in RAD51 and its paralogues (Fig 1D). The RAD51D R185Q/W constitutes a hotspot as it occurs in 100 prostate and skin samples. Another mutation occurring at higher frequency is the RAD51B L172S/W (nine patients: large intestine, prostate and soft tissue). We also checked the distribution of mutations in cancer tissues and found that most RAD51 paralogue mutations are found in prostate cancers (Fig 1E). This was not unexpected as certain paralogue polymorphisms and mutations were shown to predispose to prostate cancers [66–69]. However, when we partitioned the COSMIC samples by primary tissue, we discovered that more samples were reported for prostate cancers than for any other type of cancer (S1 Fig). Thus, the increase in mutations numbers in prostate cancers may be a consequence of more reported samples.

### 3.2. Driver mutations

Not all point mutations have an equal impact on cancer initiation and evolution. Various algorithms have been developed to classify mutations into driver and passenger [70]. Driver mutations have a high probability of causing cancer while passenger mutations accumulate as background noise. We used the CHASMPlus machine learning algorithm [51] to characterize all coding point mutations in RAD51 and its paralogues (S1 Table). The algorithm produces a probability value that can aid in the interpretation: mutations with p-values below 0.05 (significant) are considered driver while those with mutations above 0.05 (not-significant) are not. Not unexpectedly, no RAD51 mutation had a significant p-value, further suggesting that RAD51 mutations do not significantly destabilize the enzyme in human cancers. Driver mutations were also not found in RAD51B, RAD51D, XRCC2, or XRCC3. Only two driver mutations were identified in RAD51C (R24L p-value = 0.0299; P21A p-value = 0.0446). Thus, although mutations occur in the RAD51 paralogues, most are not considered significant to drive cancer, at least under the analysis undertaken here.

To visualize where these mutations map on the protein sequences, we generated cartoon diagrams of RAD51 and its paralogues (Fig 2A). RAD51 is characterized primarily by a recA domain which spans over 70% of each sequence. The recA domain contains Walker A and B domains as well as the ATP binding pocket and two DNA binding loops (L1 and L2) [71]. An N-terminal domain (NTD) was shown in yeast to be required for enhancing the activity of RAD51 and increasing stability through phosphorylation by Mec1 (human ATR) and Tel1 (human ATM) [72]. The RAD51 paralogues also have these domains except for XRCC2 which has a recA domain but not NTD. BCDX2 and CX3 complex structural analysis has also revealed that the NTD domains enhances interaction specificity between paralogues and protofilament formation [28]. Maps of truncating and high frequency mutations of RAD51 and its paralogues do not reveal any clustering of mutations in one region of any of the proteins (Fig 2A). The hotspot RAD51B L172K and RAD51D R185Q mutations occur in the recA domain. An alignment of the sequences of all proteins also shows that although the two mutations occur in the same general region, they do not correspond to the same residue (e.g., occurring in a residue in RAD51B that aligns with RAD51D) (Fig 2B).

### 3.3. Mutation classification with VEST4, Mutation Assessor and SIFT

Although CHASM can calculate the probability of a mutation causing cancer, other algorithms can also calculate the functional impact of mutations. Thus, we assessed mutations with three other algorithms hosted by OpenCravat: VEST4, Mutation Assessor, and SIFT. VEST4 predicts whether a mutation is pathogenic regardless of disease type [73], Mutation Assessor interprets the mutation based on evolutionary conservation [52], while SIFT predicts the effect of a mutation on enzyme function based on amino acid physical characteristics and sequence homology. This analysis revealed many more mutations that could affect enzyme function (S1 Table). To understand where these mutations map onto protein sequence, we generated lollipops (S2 Fig and see Materials and Methods).

VEST4 showed that many more mutations than predicted by CHASM Plus are likely to be pathogenic in all genes studied. Importantly, RAD51 had the most VEST4 mutations with a score above 0.9 (26). This shows that altering the RAD51 protein sequence is likely to cause disease. Similarly, VEST4 revealed many more pathogenic mutations in the other paralogues. The SIFT algorithm output more potentially destabilizing mutations for all genes than any of the three algorithms (S2 Fig). This was not unexpected because destabilizing the primary structure of any protein will have some effect on its function. Finally, Mutation Assessor also predicted several more mutations for each gene that are likely to have a functional impact. We did not observe any general trends in the positions of the mutations predicted with these three algorithms (e.g., clustering to one region of the protein) suggesting that enzyme function could be destabilized by having non-active site mutations. Taken together, these analyses show that many more mutations in RAD51 and its paralogues are likely to destabilize the function of the genes even though they may not be classified as “driver”. Because cancer genomes are characterized by mutations in multiple genes (over 1000), it stands to reason that combinations between less deleterious mutations in RAD51 and its paralogues with mutations in other cell cycle or genome regulating genes is likely to produce combinatorial effects that cause cellular transformation.

### 3.4. Co-occurring mutations between RAD51 and its paralogues

Because mutations in RAD51 do not appear to be inactivating, we next checked to see if the repair machinery is destabilized by co-occurring mutations between RAD51 and its paralogues. We used the cBioPortal mutual exclusivity calculator to see if there is a tendency for mutations to co-occur [55]. This analysis shows that mutations between any of the two genes studied here have a significant probability to co-occur (S2A Table). A caveat of this analysis is that it usese the cBioPortal data which is similar to COSMIC, but not identical (e.g. cBioPortal and COSMIC largely deposit the same samples but they also have some unique ones). When we counted all COSMIC samples with mutations in RAD51 that also had mutations in any of the paralogues, we found very few instances of co-mutations: out of 158 samples with RAD51 mutations 6 had a RAD51B mutation (3.8%), 6 had a RAD51C mutation (3.8%), 11 had a RAD51D mutation (7%), 5 had a XRCC2 mutation (3.2%) and 1 had a XRCC2 mutation (0.6%) (Fig 2C and S2B Table). These data suggest that most cancer samples on COSMIC do not have mutations in multiple genes withi the RAD41 and paralogues family.

### 3.5. Protein structure modeling of high-frequency mutations

**Fig 3:**
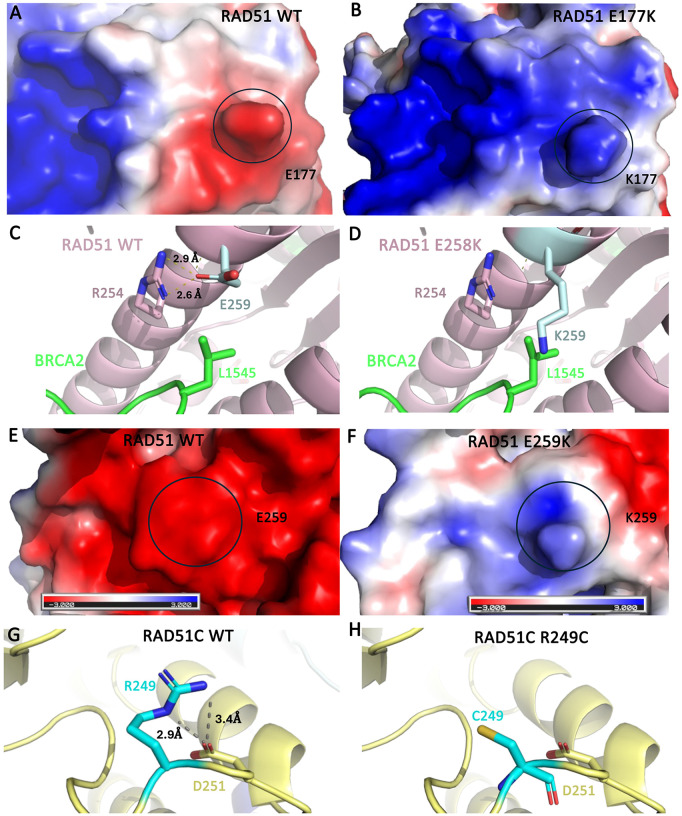
Modeling of key high-frequency mutations that affected protein structure. **A.** Electrostatic surface potential of RAD51 WT E177 (circled) from a published X-ray structure (PDB ID: 1N0W) [74]. **B.** Electrostatic surface potential of RAD51 E177K mutant (circled). An acidic surface potential is shown in red, basic in blue, and neutral in red. **C.** A cartoon representation of RAD51 is shown in pink in complex with BRCA2 in green [74]. The RAD51 residue E259 is shown in light blue sticks and R254 is shown in pink sticks, and BRCA2 residue L1545 is shown in green sticks. Polar interactions are shown with yellow dashed lines between E259 and R254, which are labeled with their respective measurements. **D.** A model of the RAD51 E259K mutant is shown with the same coloring as panel C. **E.** An electrostatic surface potential of RAD51 WT E259 (circled) in comparison to **F.** RAD51 E259K mutant. **G.** A cartoon representation of RAD51C is shown in light yellow from a published cryo-EM structure (PDB ID: 8OUZ) [75]. The RAD51 residue R249 is shown in cyan sticks and D251 is shown in yellow sticks. Polar interactions are shown with gray dashed lines between R249 and D251, which are labeled with their respective measurements. **H.** A model of the RAD51C R249C mutant is shown with the same coloring as panel G.

The high-frequency missense mutations identified in Figs 1D and 2A were modeled onto 3D protein structures to determine their impact on protein structure. Changes in polar tertiary or quaternary structure interactions, electrostatic surface potential, and protein structural stability were analyzed (S3–S12 Figs and S3 Table). The RAD51 mutations were modeled onto an X-ray structure of the RAD51-BRCA2 BRC repeat complex (PDB ID: 1N0W) (S3 and S8 Figs) [74]. The RAD51 E177K mutation was found to drastically change the local electrostatic surface potential (Fig 3A and 3B) and disrupt a polar tertiary structure interaction (Fig 3C and 3D). This residue is also located near the interface with BRCA2 and this mutation could disrupt this interaction. The RAD51 E259K mutation also resulted in large changes to the local electrostatic surface potential of the protein (Fig 3E and 3F) and is predicted by CUPSAT to destabilize the protein structure by 1.1 kcal/mol. All of the high-frequency RAD51 missense mutations were predicted to be destabilizing and the E259D had the largest ΔΔG of −2.0 kcal/mol.

To model the RAD51B high-frequency mutations at residue 172, an AlphaFold model was used [60] since this residue was not resolved in published X-ray or cryo-EM structures (S4 and S9 Figs). Neither of these mutations were observed to have a significant impact on tertiary structure interactions, electrostatics, or protein stability.

To model the RAD51C high-frequency missense mutations, a cryo-EM structure of the BCDX2 complex (RAD51B-RAD51C-RAD51D-XRCC2) (PDB ID: 8OUZ) was used to model the P21 and R249 mutations (S5 and S10 Figs) [28]. Residue 368 was not resolved in this cryo-EM structure, so an AlphaFold model of RAD51C was used to model the mutations as this position [60]. The P21S/T mutations did not impact tertiary structure interactions or electrostatics. Of the mutations modeled, R249C (Fig 3G and 3H) and R249H were both found to disrupt a polar tertiary structure interaction. The R249H mutation also changed the local electrostatic surface potential of the protein. The two R368 mutations did not affect tertiary structure interactions but did change the local electrostatics. CUPSAT predicted five out of six of the RAD51C mutations to destabilize the protein structure, with R368W being the most destabilizing with a ΔΔG of –5.9 kcal/mol. The R368Q mutation was the only stabilizing mutation with a ΔΔG of +1.03 kcal/mol.

Another cryo-EM structure of the BCDX2 complex (PDB ID: 8FAZ) was used to model RAD51D mutations (S6 and S11 Figs) [28]. Both R185 mutations decreased tertiary structure interactions and altered the electrostatic surface potential of the protein. Both were found to be destabilizing to the protein structure by CUPSAT but the R185W mutation had a larger impact than R185Q.

No experimentally determined structures of human XRCC3 have been published, so an AlphaFold model was used to model the high-frequency missense mutations (S7 and S12 Figs). The two mutations observed as H183 did not impact tertiary structure interactions or local electrostatics of the protein. However, both were predicted to destabilize the protein structure by CUPSAT with ΔΔG values of −1.55 and -2.61 kcal/mol for H183N and H183P, respectively.

Overall, the structural analyses demonstrates that the majority high-frequency mutations in RAD51, RAD51C, and RAD51D strongly impacted the protein structure. All of the RAD51 mutations modeled were predicted to be destabilizing to the overall protein structure by CUPSAT. The E177K RAD51 mutation impacted protein structure in all three metrics analyzed and could impact the interaction with BRCA2. In RAD51C, the R368W mutation was the most destabilizing mutation of all analyzed in all proteins. In addition, it was observed that R249C disrupted tertiary structure interactions and the electrostatic surface potential. Both RAD51D high-frequency mutations effected protein structure in all three metrics. Whereas RAD51B mutations did not impact protein structure and the XRCC3 mutations only had a mild impact.

### 3.6. Expression and selection pressure

**Fig 4:**
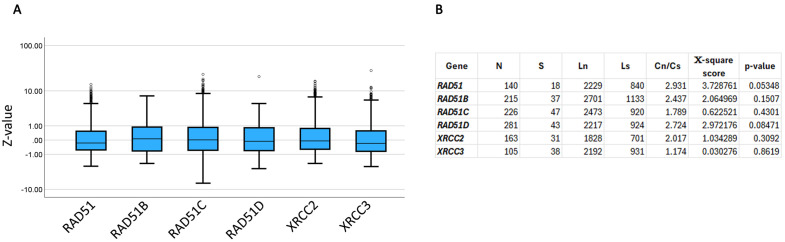
Gene expression and selection pressure. **A**. Gene expression for The Genome Cancer Atlas (TCGA) samples. The data show pan-cancer Z-values distribution for each gene. The Y-axis is logarithmic for better visualization. **B**. Selection pressure for RAD51 and its paralogues. Selection pressure was calculated as descried in [62].

Another hallmark of cancer is changes in gene expression. COSMIC reports gene expression values for TCGA samples which represents a subset of the data queried here because mutation is reported for both TCGA and non-TCGA samples. Gene expression is given in Z-values which are interpreted as normal between −2 and 2. Values below −2 are indicative of under-expression and those above 2 of over-expression [61]. A pan-cancer analysis of gene expression for RAD51 and its paralogues shows that they are within the normal range (Fig 4A and S4 Table). Thus, changes in expression of one of the paralogues is unlikely to be a mechanism for compensating for mutations in RAD51.

If a mutation provides an advantage to a cancer cell, it will undergo selective pressure and be statistically significantly more represented in cancer patients. To calculate selective pressure in the genes studied here, we employed a common statistical analysis that can reveal positive, neutral, or negative selection [62]. This analysis shows that all genes are under neutral selection as the probability values are insignificant (Fig 4B and S5 Table). Therefore, mutations in RAD51 and its paralogues are not biased by selective pressure.

### 3.7. Comparisons of mutations reported on COSMIC with those reported on ClinVar

NCBI ClinVar reports multiple germline mutations in RAD51 and its paralogues (S6 Table). ClinVar mutations have been categorized as benign, pathogenic, likely pathogenic, uncertain significance and conflicting classification of pathogenicity. We extracted all the pathogenic, likely pathogenic, and conflicting classification of pathogenicity for all genes and compared them with those reported on COSMIC (Tables 1 and S6). This analysis identified several germline mutations that are also reported on COSMIC including some of the high frequency mutations (bold in Table 1). Thus, some of the mutations reported on COSMIC may have been inherited and are not somatic.

**Table 1:** Mutations reported on both COSMIC and ClinVar.

Gene	^1^COSMIC	^2^ClinVar	^3^ClinVar Pathogenic	^4^Co-occurrence	^5^Coordinates of mutations reported on both sites
**RAD51**	141	272	14	4	^**6**^**E259K**, R251Q, R255*, R151Q
**RAD51B**	239	332	6	2	A76T, V207L
**RAD51C**	249	1285	612	63	**P21A/S**, R249C, A152T, A175T, A308G, A354T, A55V, D13N, D171E, E154G, E161DE374K, E45D, E89K, G112A, G264S, G77D, H187Q, K26M, K83N, L230F, L257V, L262V, L38F, L39V, M136L, P18S, P247L, P330S, R12Q, R193Q, R212H, R214C, **R249H**, R258H R292K, R312W, R319Q, R366Q, **R368W**, R7C, S362F, S53C, S79C, T102I, T349A, T5M, V151M, V169A, V223I, V351I, E67*, G149*, R237*, R319*, S105fs, S105fs, Y210*, A76fs, Q178*, Q285*, Y210*, Q344R, G149fs
**RAD51D**	505	1075	275	28	N158H, A210T, A313V, G345C, G309S, R286C, R252*, R320*, R165H, V152I, E121*, A230V, F193L, **R185W**, V224I, R252*, R273*, S227L, R185Q, A68T, M16T, R108C, S46C, Q41*, R100*, M312I, M180I, M292I, Q339H,
**XRCC2**	185	549	14	1	**L117fs**
**XRCC3**	135	69	0	0	

^1^COSMIC total samples reported.

^2^ClinVar total samples reported.

^3^Includes ClinVar mutations reported as pathogenic, likely pathogenic and of uncertain significance (conflicting classification).

^4^ClinVar pathogenic mutations that are also reported on COSMIC.

^5^Coordinates of the ClinVar pathogenic mutations that are reported on COSMIC.

^6^Mutations in **bold** were also identified as frequent (see Fig 2).

RAD51C had the most reported germline mutations (Table 1). A recent report investigated the effect on HR of some of these mutations [46] and found that they range from no effect (e.g., WT levels) to almost complete abrogation of HR. They’ve also identified that most RAD51C mutations that disrupt HR also disrupt interaction with other HR paralogues and a cluster of mutations affect DNA binding and ATP hydrolysis.

In this report, we did not follow our analysis with experimental validation which limits our interpretation of these findings. However, given the previous analysis of RAD51C mutations, we suspect that some of these mutations will affect HR. Therefore, further validation of these data is necessary to understand which mutations are likely to destabilize the repair machinery and produce chromosomal instability phenotypes.

## Conclusion

Here, we used in silico protein structure and machine learing algorithms to investigate the potential effects of cancer mutations in RAD51 and its paralogues. We identified many more predicted deleterious mutations in the RAD51B and RAD51C paralogues than RAD51 suggesting that destabilizing the paralogues may be sufficient to affect HR. Indeed, as mentioned above, certain RAD51C mutations can abrogate HR without mutations in the other RAD51 paralogues [46]. The structural modeling showed that high-frequency mutations in RAD51, RAD51C, and RAD51D strongly affected protein structure whereas the high-frequency mutations in RAD51B and XRCC3 did not. Thus, an apparent lack of mutation in RAD51 in cancer cells may not necessarily mean that the repair machinery is not destabilized. This may also explain the “RAD51 paradox”: mutations in paralogues are sufficient to affect HR. Although, our study only provides an in silico analysis and would need to be validated by experiments, these findings nevertheless highlight how mutations in multiple components of the HR machinery work together to destabilize repair.

## Supporting information

S1 Fig Samples reported on COSMIC partitioned by primary cancer. (PDF)

S2 Fig Lollipops showing mutations predicted pathogenic or having a functional impact using SIFT, MutationAssessor, and VEST4. (PDF)

S3 Fig Polar tertiary structure interactions for high-frequency mutations in RAD51A. (PDF)

S4 Fig Polar tertiary structure interactions for high-frequency mutations in RAD51B. (PDF)

S5 Fig Polar tertiary structure interactions for high-frequency mutations in RAD51C. (PDF)

S6 Fig Polar tertiary structure interactions for high-frequency mutations in RAD51D. (PDF)

S7 Fig Polar tertiary structure interactions for high-frequency mutations in XRCC3. (PDF)

S8 Fig Electrostatic surface potential calculations in RAD51A. (PDF)

S9 Fig Electrostatic surface potential calculations in RAD51B. (PDF)

S10 Fig Electrostatic surface potential calculations in RAD51C. (PDF)

S11 Fig Electrostatic surface potential calculations in RAD51D. (PDF)

S12 Fig Electrostatic surface potential calculations in XRCC3. (PDF)

S1 Table COSMIC mutation files and OpenCravat algorithms outputs. (XLSX)

S2 Table Mutation co-occurrence between the genes studied here. (XLSX)

S3 Table Summary of structural analysis results of co-occurring and high-frequency mutations. (DOCX)

S4 Table Pan-cancer expression profiles for RAD51 and its paralogues shown as Z-values. (XLSX)

S5 Table Calculation of selection pressure for RAD51 and its paralogues. (XLSX)

S6 Table Comparisons of COSMIC mutations with those reported on ClinVar. (XLSX)
