# Supplementary figures and images for: Cancer mutations in RAD51 and its paralogues
Source: PLoS One. 2026 May 14;21(5):e0349105. doi: 10.1371/journal.pone.0349105 (PMC13175330; doi:10.1371/journal.pone.0349105)

Supplemental Figure 1. Samples reported on COSMIC partitioned by primary cancer

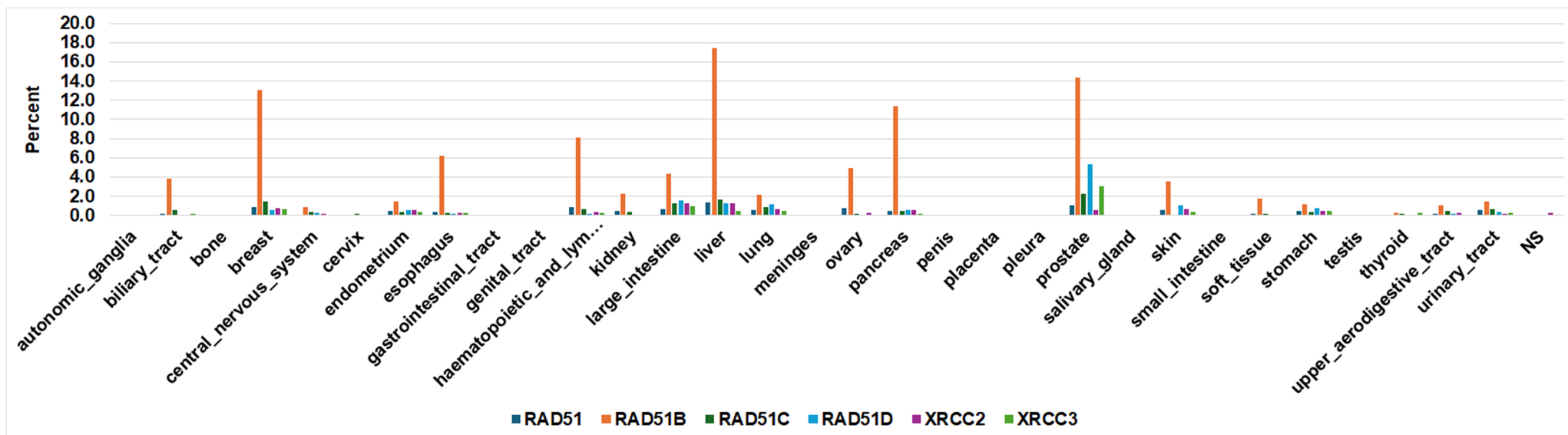

Supplement: S1 Fig — (PDF) [file pone.0349105.s001.pdf]
