# Supplementary material for: Cancer mutations in RAD51 and its paralogues
Source: PLoS One. 2026 May 14;21(5):e0349105. doi: 10.1371/journal.pone.0349105 (PMC13175330; doi:10.1371/journal.pone.0349105)

Supplemental Figure 2. Lollipops showing SIFT, MutationAssesor and VEST4 predicted pathogenic or having a functional impact.

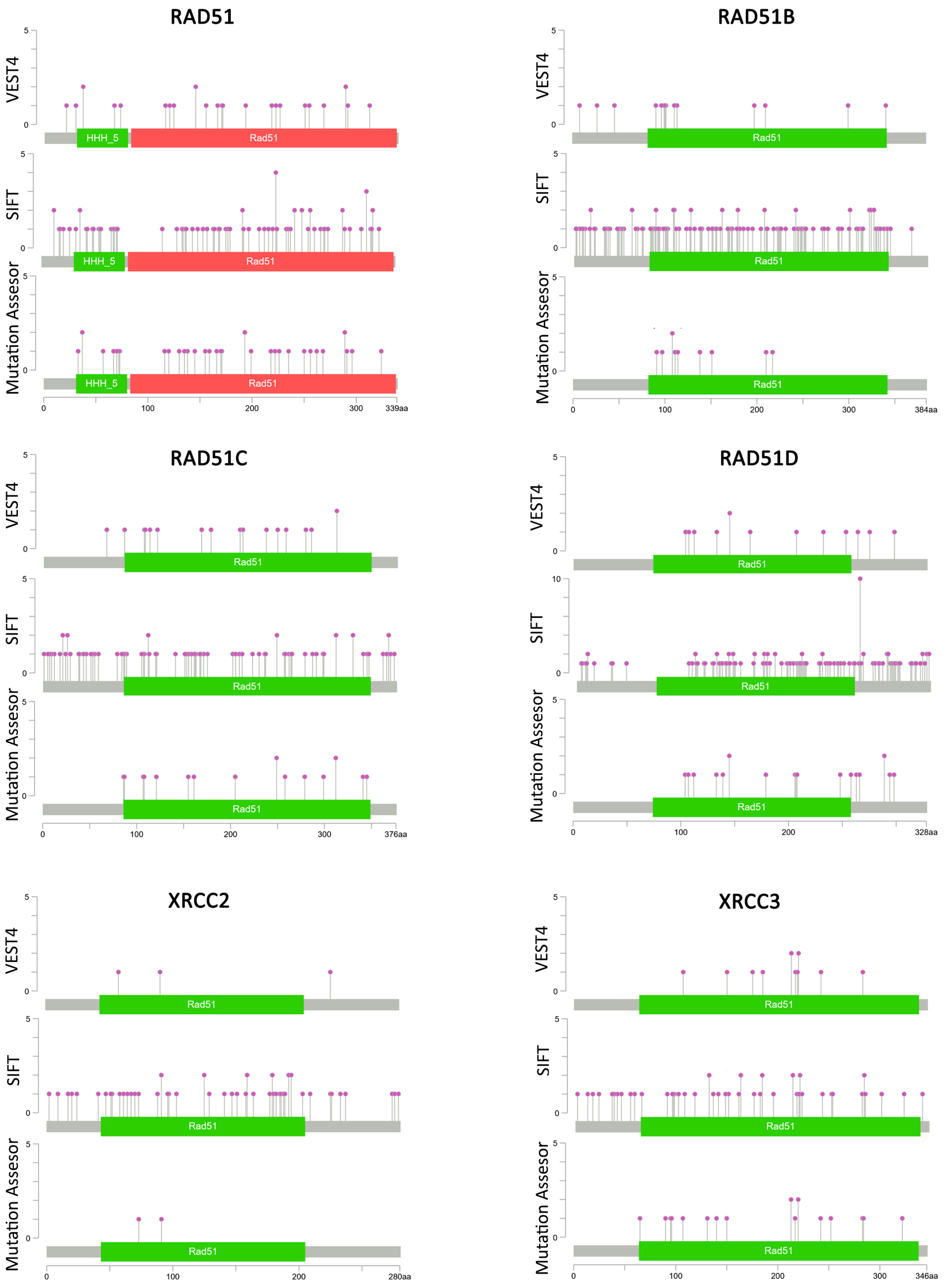

Supplement: S2 Fig — (PDF) [file pone.0349105.s002.pdf]
