# Supplementary material for: Cancer mutations in RAD51 and its paralogues
Source: PLoS One. 2026 May 14;21(5):e0349105. doi: 10.1371/journal.pone.0349105 (PMC13175330; doi:10.1371/journal.pone.0349105)

Supplemental Figure 3. Polar tertiary structure interactions for high-frequency mutations in RAD51A. High-frequency mutations were mapped onto an X-ray structure of RAD51 bound to BRCA2 (PDB ID: 1N0W). RAD51A is shown as a pink cartoon and BRCA2 in green. The residue of interest is shown in light blue sticks and nearby residues on RAD51A are shown in pink sticks or green sticks for BRCA2. Polar interactions are shown with yellow dashed lines with their respective measurements in angstroms.

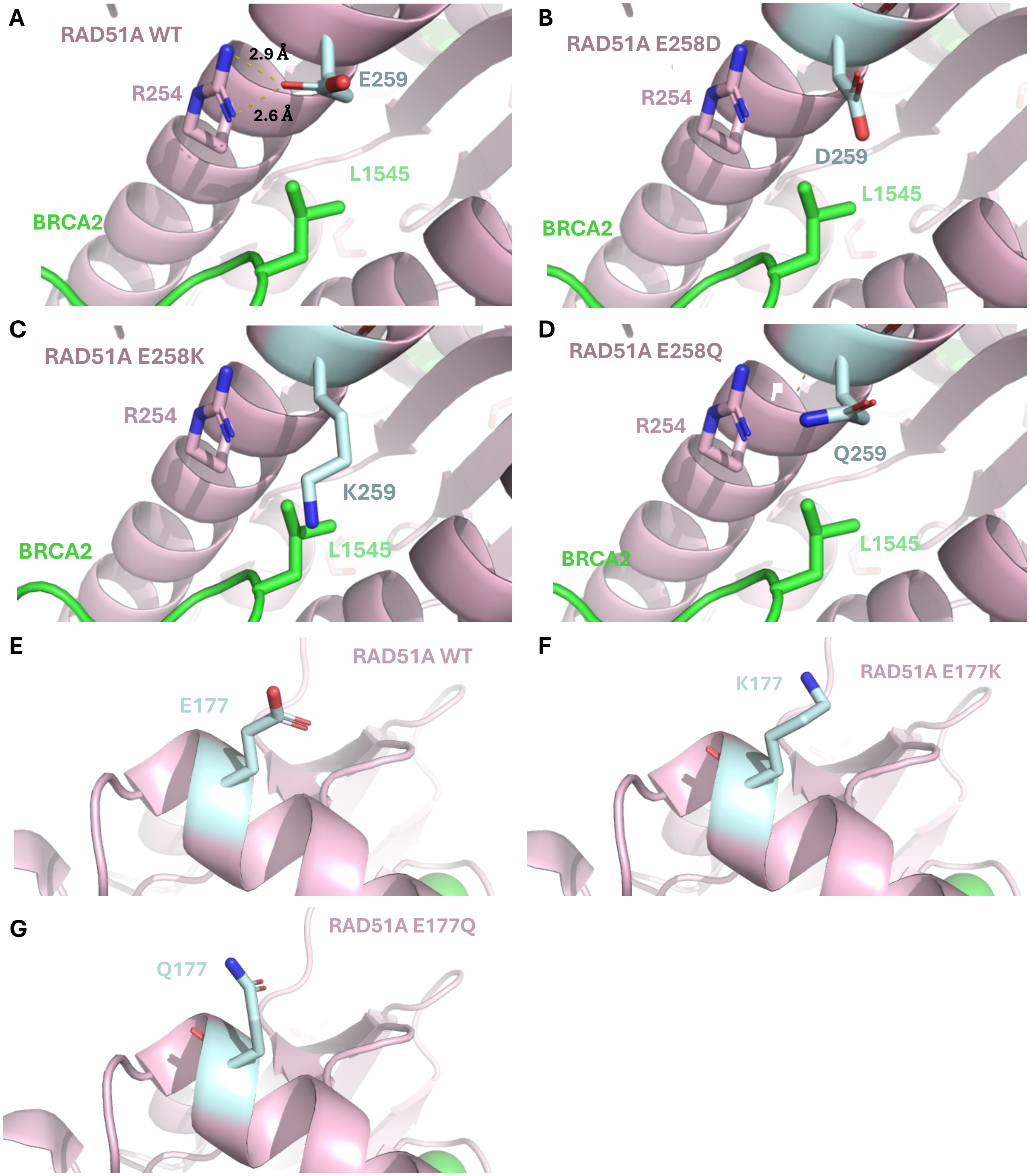

Supplement: S3 Fig — (PDF) [file pone.0349105.s003.pdf]
