# Supplementary material for: Cancer mutations in RAD51 and its paralogues
Source: PLoS One. 2026 May 14;21(5):e0349105. doi: 10.1371/journal.pone.0349105 (PMC13175330; doi:10.1371/journal.pone.0349105)

Supplemental Figure 4. Polar tertiary structure interactions for high-frequency mutations in RAD51B. High-frequency mutations were mapped onto an AlphaFold structure of RAD51B. RAD51B is shown as a gray cartoon. The residue of interest is shown in light green sticks and nearby residues are shown in cyan sticks. Polar interactions are shown with yellow dashed lines.

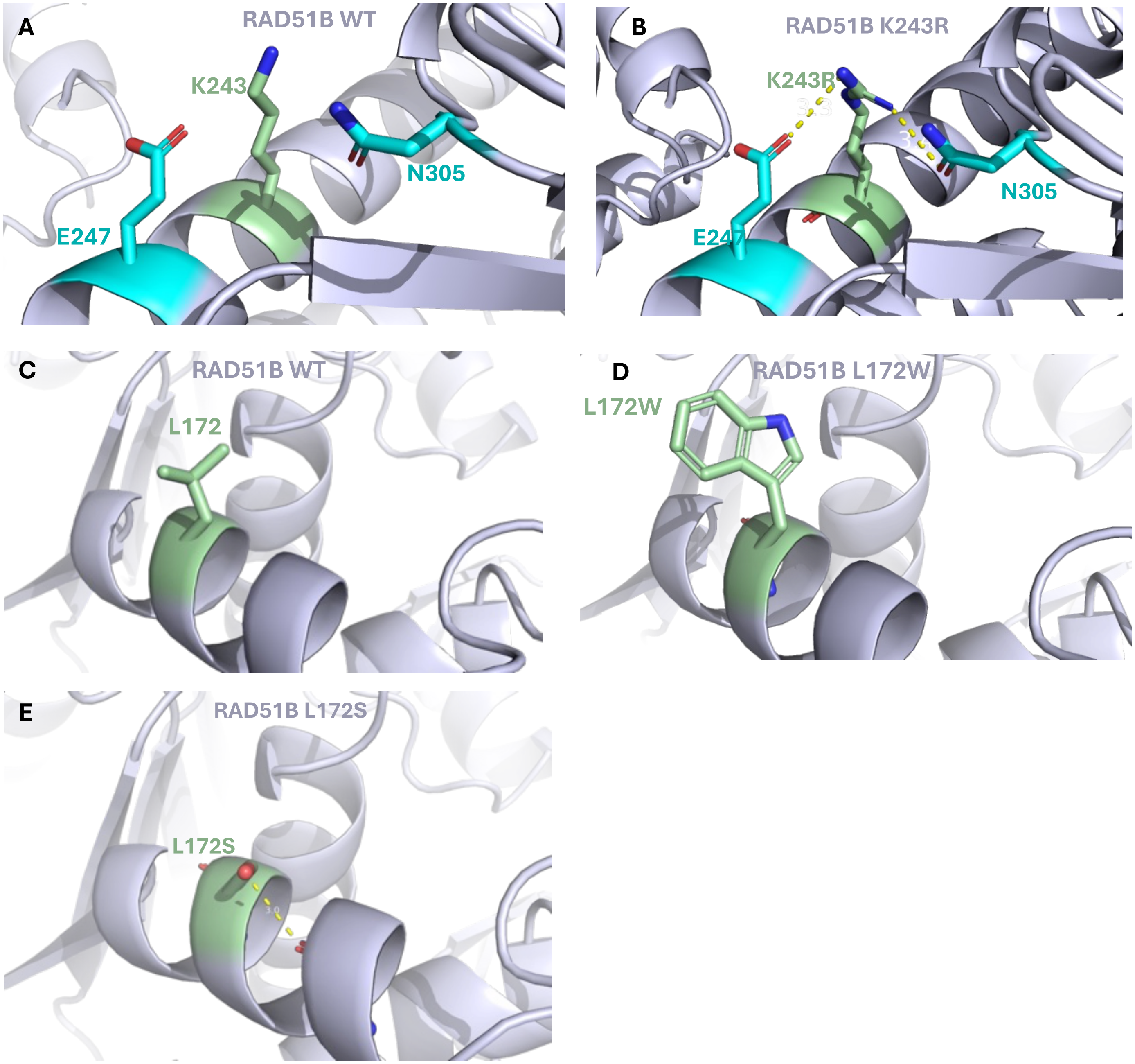

Supplement: S4 Fig — (PDF) [file pone.0349105.s004.pdf]
