# Supplementary material for: Cancer mutations in RAD51 and its paralogues
Source: PLoS One. 2026 May 14;21(5):e0349105. doi: 10.1371/journal.pone.0349105 (PMC13175330; doi:10.1371/journal.pone.0349105)

Supplemental Figure 5. Polar tertiary structure interactions for high-frequency mutations in RAD51C. High-frequency mutations were mapped onto a cryo-EM structure of the RAD51B-RAD51C-RAD51D-XRCC2 complex (PDB ID: 8OUZ). RAD51B is shown as a purple cartoon and RAD51C is shown in yellow. The residue of interest for RAD51C is shown in cyan sticks. Nearby residues are shown as purple sticks for RAD51B. Polar interactions are shown with gray dashed lines.

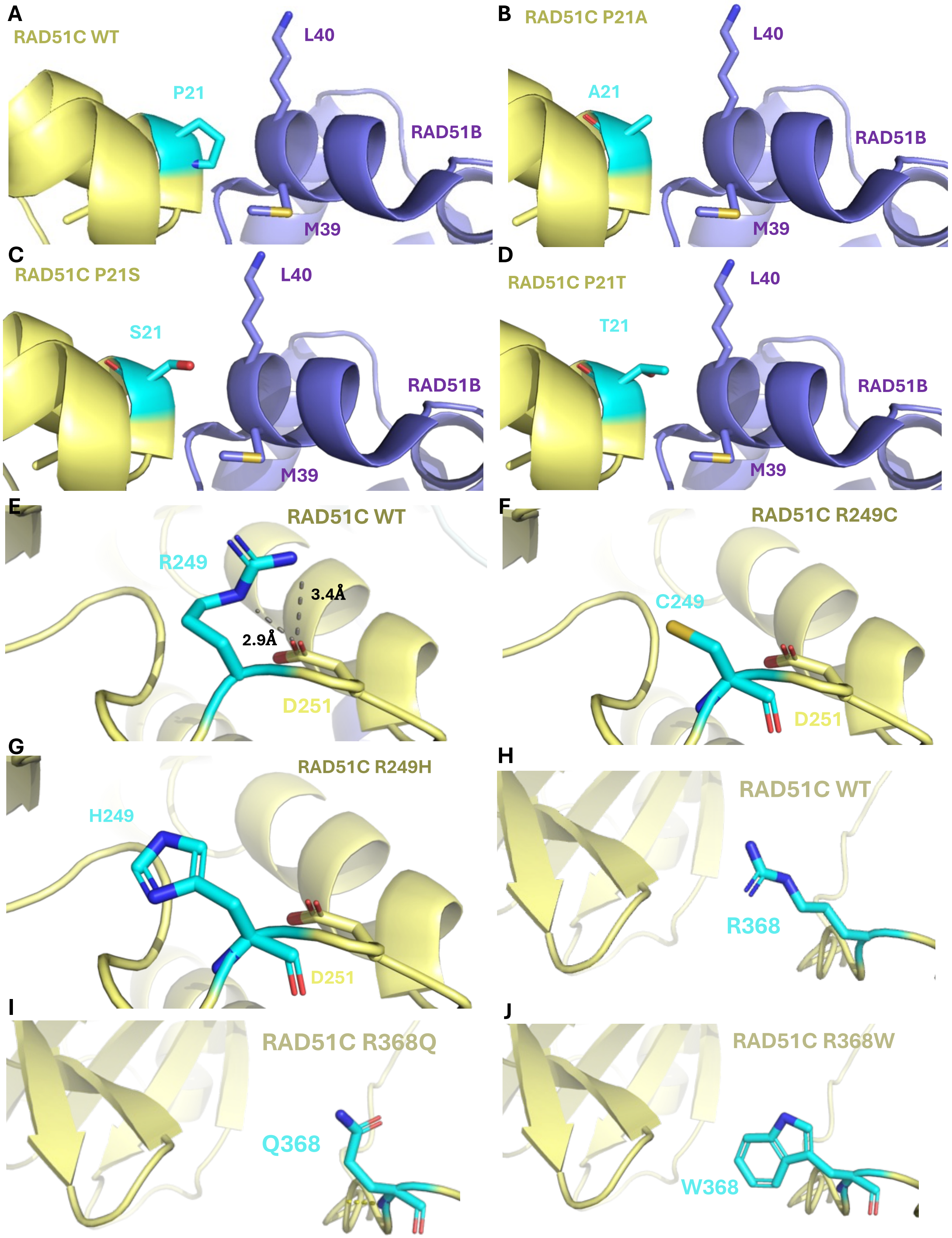

Supplement: S5 Fig — (PDF) [file pone.0349105.s005.pdf]
