# Supplementary material for: Cancer mutations in RAD51 and its paralogues
Source: PLoS One. 2026 May 14;21(5):e0349105. doi: 10.1371/journal.pone.0349105 (PMC13175330; doi:10.1371/journal.pone.0349105)

Supplemental Figure 6. Polar tertiary structure interactions for high-frequency mutations in RAD51D. High-frequency mutations were mapped onto a cryo-EM structure of the RAD51B-RAD51C-RAD51D-XRCC2 complex (PDB ID: 8OUZ). RAD51C is shown as a yellow cartoon and RAD51D is shown in pale cyan. The residue of interest for RAD51D is shown in pink sticks. Nearby residues are shown as pale cyan sticks for RAD51D.

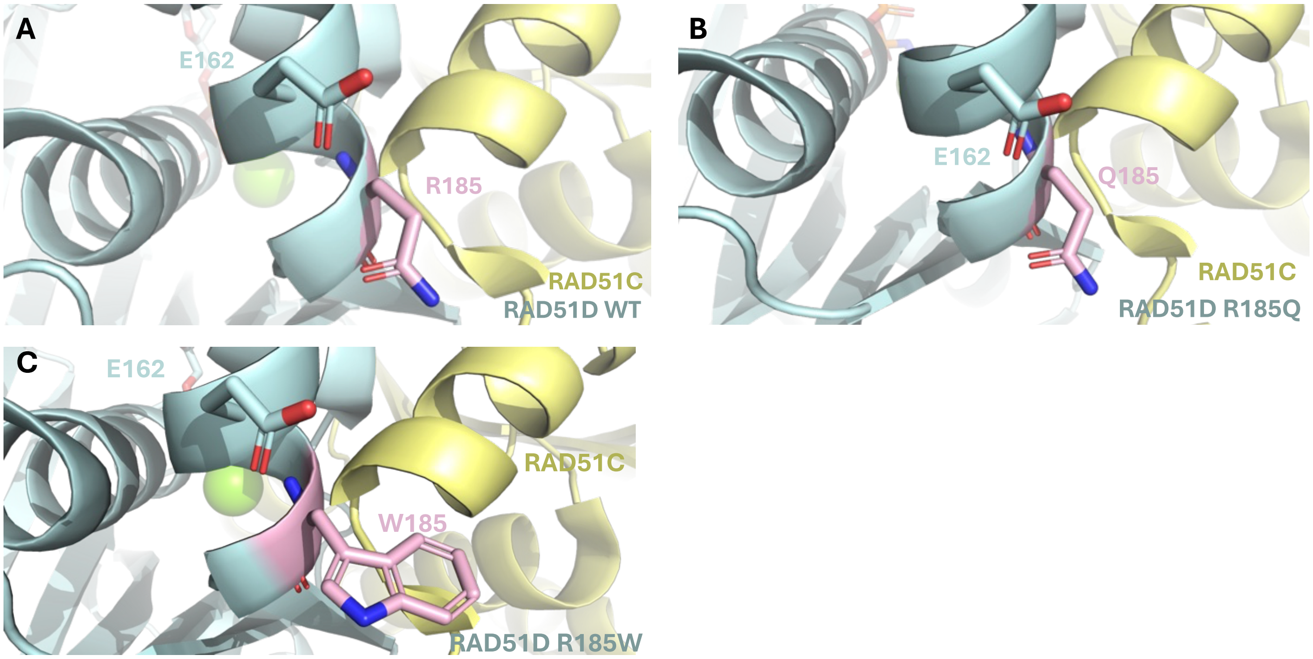

Supplement: S6 Fig — (PDF) [file pone.0349105.s006.pdf]
