# Supplementary material for: Cancer mutations in RAD51 and its paralogues
Source: PLoS One. 2026 May 14;21(5):e0349105. doi: 10.1371/journal.pone.0349105 (PMC13175330; doi:10.1371/journal.pone.0349105)

Supplemental Figure 7. Polar tertiary structure interactions for high-frequency mutations in XRCC3. High-frequency mutations were mapped onto an AlphaFold structure of XRCC3. XRCC3 is shown as a purple cartoon. The residue of interest is shown in pink sticks.

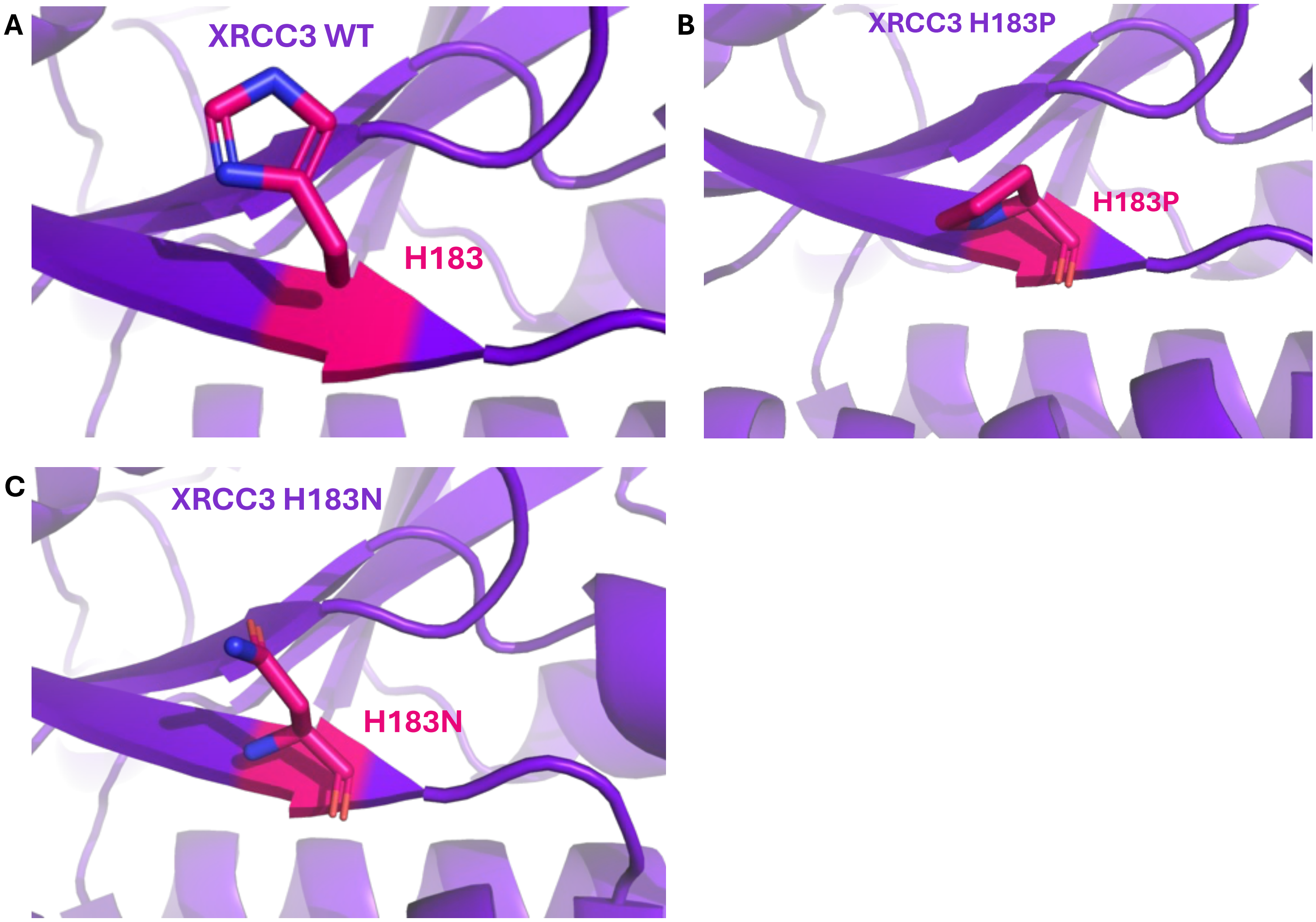

Supplement: S7 Fig — (PDF) [file pone.0349105.s007.pdf]
