# Supplementary material for: Cancer mutations in RAD51 and its paralogues
Source: PLoS One. 2026 May 14;21(5):e0349105. doi: 10.1371/journal.pone.0349105 (PMC13175330; doi:10.1371/journal.pone.0349105)

Supplemental Figure 8. Electrostatic surface potential calculations in RAD51A. High-frequency mutations were mapped onto an X-ray structure of RAD51 bound to BRCA2 (PDB ID: 1N0W). Electrostatic surface potentials are shown as red, blue, and white for acidic, basic, and neutral areas of the protein, respectively. The location of the mutated residue is shown with a black circle.

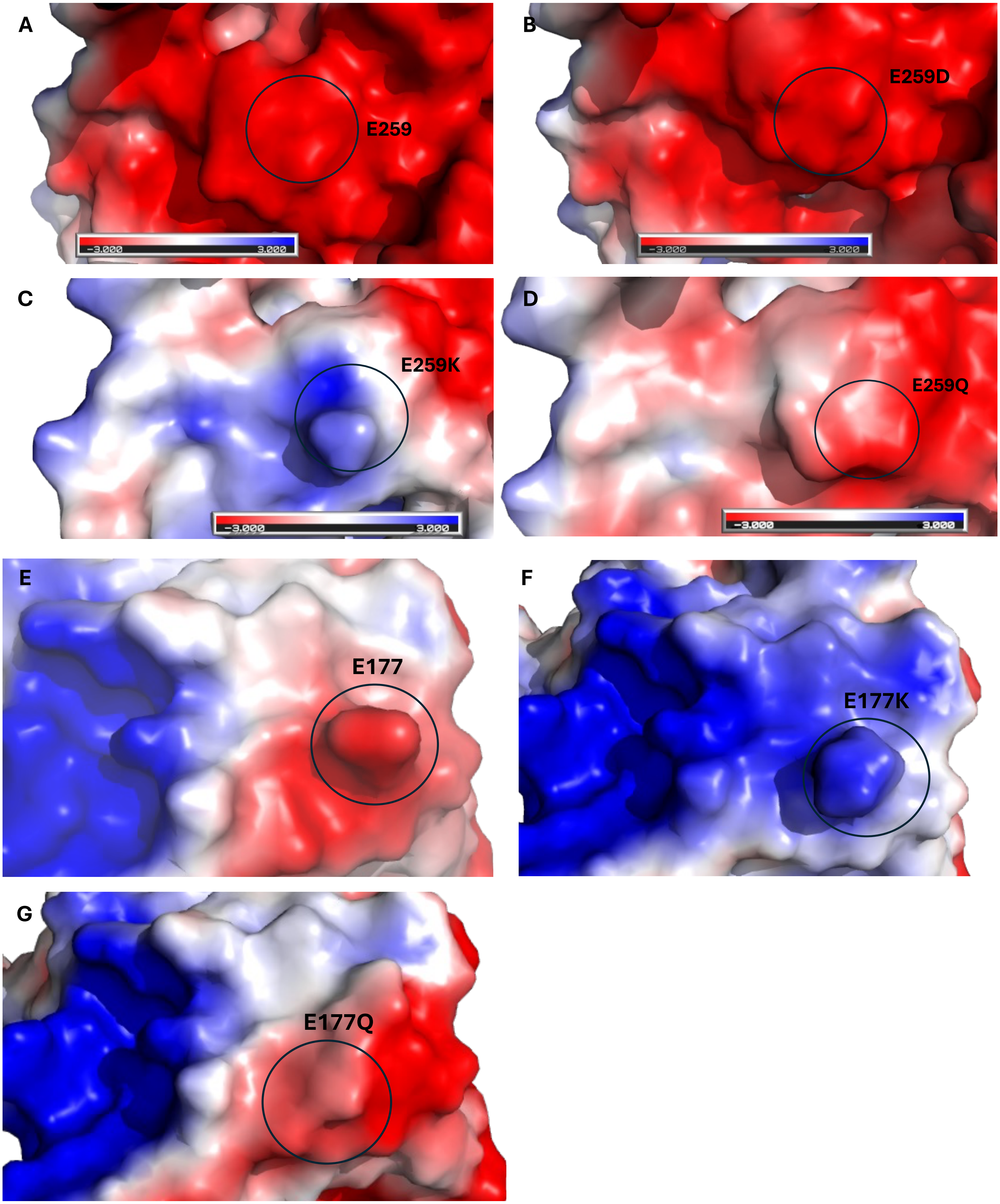

Supplement: S8 Fig — (PDF) [file pone.0349105.s008.pdf]
