# Supplementary material for: Cancer mutations in RAD51 and its paralogues
Source: PLoS One. 2026 May 14;21(5):e0349105. doi: 10.1371/journal.pone.0349105 (PMC13175330; doi:10.1371/journal.pone.0349105)

Supplemental Figure 9. Electrostatic surface potential calculations in RAD51B. High-frequency mutations were mapped onto an AlphaFold structure of RAD51B. Electrostatic surface potentials are shown as red, blue, and white for acidic, basic, and neutral areas of the protein, respectively. The location of the mutated residue is shown with a black circle.

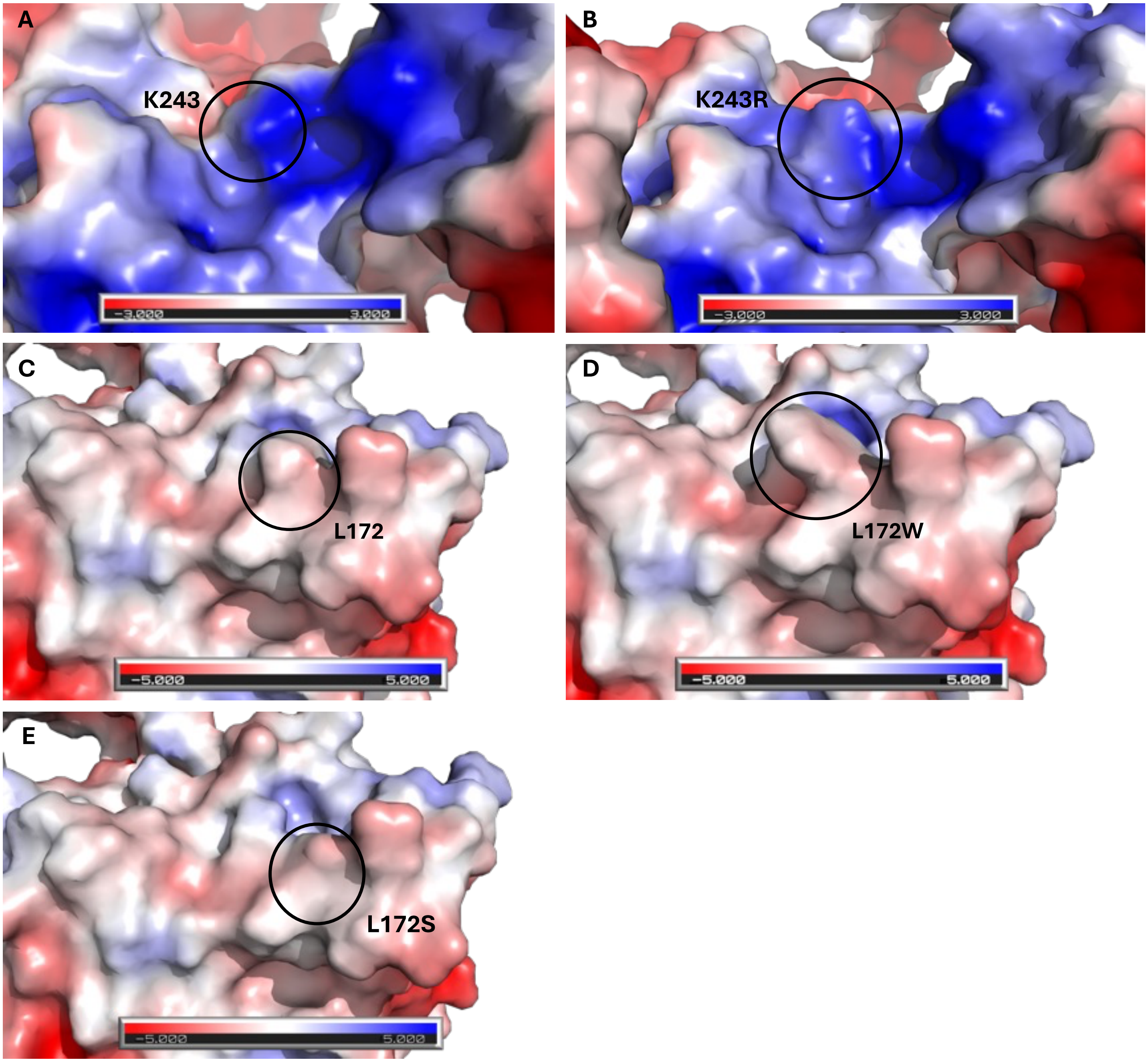

Supplement: S9 Fig — (PDF) [file pone.0349105.s009.pdf]
