# Supplementary material for: Cancer mutations in RAD51 and its paralogues
Source: PLoS One. 2026 May 14;21(5):e0349105. doi: 10.1371/journal.pone.0349105 (PMC13175330; doi:10.1371/journal.pone.0349105)

Supplemental Figure 11. Electrostatic surface potential calculations in RAD51D. High-frequency mutations were mapped onto a cryo-EM structure of the RAD51B-RAD51C-RAD51D-XRCC2 complex (PDB ID: 8OUZ). Electrostatic surface potentials are shown as red, blue, and white for acidic, basic, and neutral areas, respectively. The location of the mutated residue is shown with a black circle.

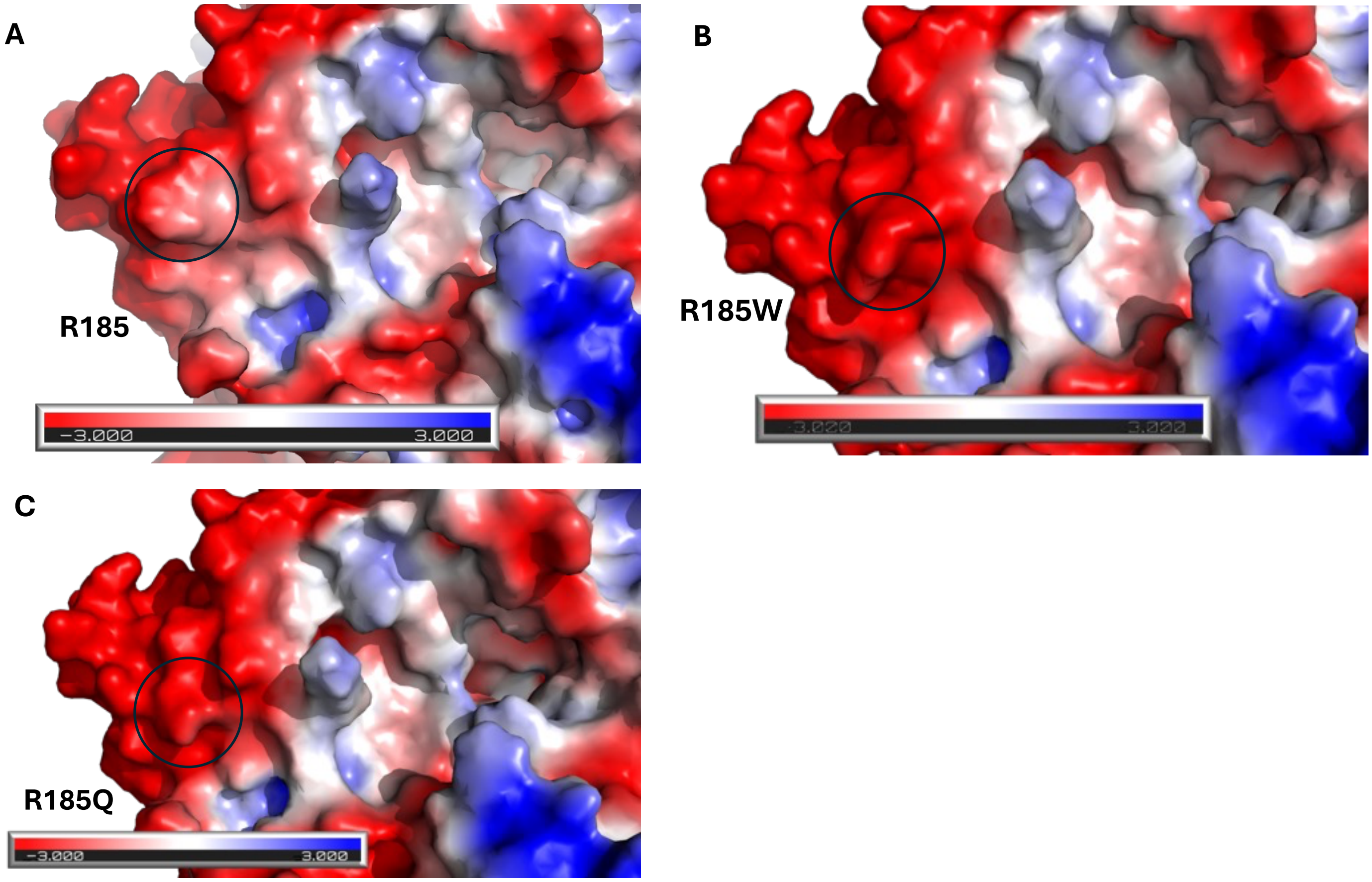

Supplement: S11 Fig — (PDF) [file pone.0349105.s011.pdf]
