# Supplementary material for: Cancer mutations in RAD51 and its paralogues
Source: PLoS One. 2026 May 14;21(5):e0349105. doi: 10.1371/journal.pone.0349105 (PMC13175330; doi:10.1371/journal.pone.0349105)

Supplemental Figure 12. Electrostatic surface potential calculations in XRCC3. High-frequency mutations were mapped onto an AlphaFold structure of XRCC3. Electrostatic surface potentials are shown as red, blue, and white for acidic, basic, and neutral areas, respectively. The location of the mutated residue is shown with a black circle.

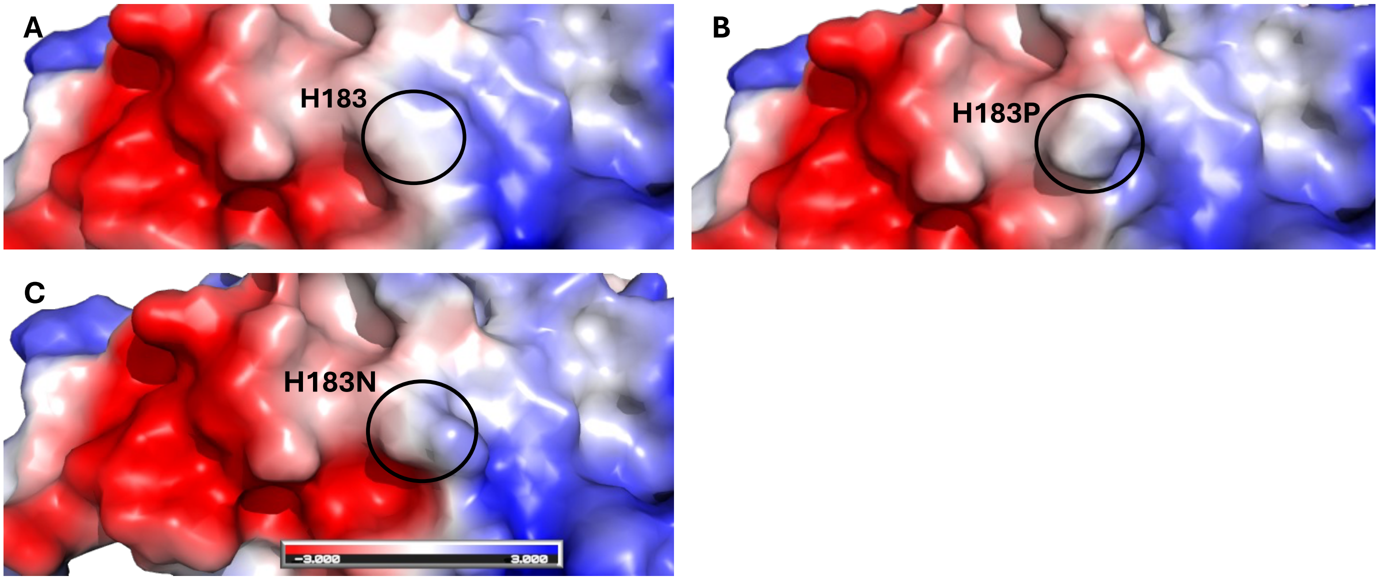

Supplement: S12 Fig — (PDF) [file pone.0349105.s012.pdf]
