# Supplementary material for: Cancer mutations in RAD51 and its paralogues
Source: PLoS One. 2026 May 14;21(5):e0349105. doi: 10.1371/journal.pone.0349105 (PMC13175330; doi:10.1371/journal.pone.0349105)
Supplement: S3 Table — (DOCX) [file pone.0349105.s015.docx]

**Supplemental Table 3. Summary of structural analysis results of co-occurring and high-frequency mutations.**

|  | | **PyMOL Analysis** | | **CUPSAT Analysis** | | | |
| --- | --- | --- | --- | --- | --- | --- | --- |
| **Protein** | **Mutation** | **Changes in Polar Interactions?** | **Changes in Electrostatic Surface Potential?** | **Predicted ∆∆G Value (kcal/mol)** | **Torsion Favorability** | **Overall Stability** | **Structure Source** |
| RAD51 | E177K | No | Acidic to Basic | -0.46 | Favorable | Destabilizing | AlphaFold |
| RAD51 | E177Q | No | No Change | -0.42 | Favorable | Destabilizing | AlphaFold |
| RAD51 | E259D | Yes, Decrease | No Change | -2.0 | Unfavorable | Destabilizing | AlphaFold |
| RAD51 | E259K | Yes, Decrease | Acidic to Basic | -1.1 | Favorable | Destabilizing | AlphaFold |
| RAD51 | E259Q | Yes, Decrease | Acidic to Neutral | -1.03 | Favorable | Destabilizing | AlphaFold |
| RAD51B | L172S | Yes, Increase | No Change | -0.94 | Unfavorable | Destabilizing | AlphaFold |
| RAD51B | L172W | No | No Change | -0.80 | Favorable | Destabilizing | AlphaFold |
| RAD51C | P21A | No | No Change | -1.0 | Favorable | Destabilizing | AlphaFold |
| RAD51C | P21S | No | No Change | -1.68 | Unfavorable | Destabilizing | AlphaFold |
| RAD51C | P21T | No | No Change | -1.09 | Unfavorable | Destabilizing | AlphaFold |
| RAD51C | R249C | Yes, Decrease | No Change | -2.99 | Unfavorable | Destabilizing | AlphaFold |
| RAD51C | R249H | Yes, Decrease | Basic to Neutral | -0.44 | Favorable | Destabilizing | AlphaFold |
| RAD51C | R368Q | No | Basic to Neutral | +1.03 | Unfavorable | Stabilizing | AlphaFold |
| RAD51C | R368W | No | Basic to Acidic | -5.9 | Unfavorable | Destabilizing | AlphaFold |
| RAD51D | R185Q | Yes, Decrease | Slightly Acidic to Acidic | -0.13 | Favorable | Destabilizing | AlphaFold |
| RAD51D | R185W | Yes, Decrease | Slightly Acidic to Acidic | -1.29 | Favorable | Destabilizing | AlphaFold |
| XRCC3 | H183N | No | No Change | -1.55 | Unfavorable | Destabilizing | AlphaFold |
| XRCC3 | H183P | No | No Change | -2.61 | Unfavorable | Destabilizing | AlphaFold |
